# ﻿A new species of *Sinocyclocheilus* (Cypriniformes, Cyprinidae) from the Beipan-Jiang in Guizhou, China

**DOI:** 10.3897/zookeys.1238.136371

**Published:** 2025-05-13

**Authors:** Leishan Wang, Qi Luo, Renyi Zhang

**Affiliations:** 1 School of Life Sciences, Guizhou Normal University, Universities Town, Huaxi District, Guiyang, Guizhou, China Guizhou Normal University Guiyang China

**Keywords:** Cavefish, karst, molecular phylogenetic analysis, morphology, new record, *Sinocyclocheiluszhenningensis*, taxonomy

## Abstract

*Sinocyclocheiluszhenningensis***sp. nov.** from the Dabang-He of the Beipan-Jiang Basin in Zhenning County, Guizhou Province, China, is described based on morphological and molecular evidence. The new species can be distinguished morphologically from all congeners in this genus by: (1) normal eyes (5.8%–8.2% SL), presence of pigmentation, with a humpback and no horn structure; (2) pelvic fin rays ii-8, dorsal-fin rays iii-8, stiff and stout last unbranched ray; (3) body depth normal (26.5%–30.1% SL) and covered with irregular black spots, body scaled, but scales buried beneath the skin; (4) lateral line complete, slightly curved ventrally, 42–47 lateral line pored scales; and (5) pectoral fin short, not reaching the anterior base of pelvic fin. Based on the mitochondrial cytochrome *b* (*Cytb*) gene phylogenetic tree, *S.zhenningensis***sp. nov.** is strongly supported as sister to the *S.cyphotergous*-*S.multipunctatus* subclade. The minimum genetic distance between *Sinocyclocheiluszhenningensis***sp. nov.** and its congeneric species is 3.8%.

## ﻿Introduction

The cyprinid genus *Sinocyclocheilus* Fang, 1936 is a representative group of cavefish endemic in China, and also the largest genus of cavefish in the world (Zhao and Zhang 2009; Ma et al. 2019; Mao et al. 2022; Luo et al. 2023; Xu et al. 2023; Shao et al. 2024). It is so far found in the karstic areas of southern China, encompassing Guizhou, Yunnan, Guangxi, Hubei and Hunan provinces and Chongqing City, drained by rivers flowing into the mid-upper Yangtze River and Pearl River basins (Zhao and Zhang 2006, 2009; Jiang et al. 2019; Mao et al. 2021; Luo et al. 2023). Eighty species of *Sinocyclocheilus* have been recognized as valid, 17 of which are found in Guizhou Province (Luo et al. 2023; Xu et al. 2023; Fan et al. 2024; Shao et al. 2024). Only four species are harbored in the Beipan-Jiang of the Pearl River Basin.

The *S.cyphotergous* group, one of four groups delineated by Zhao and Zhang (2009) within the genus *Sinocyclocheilus*, initially comprised two species: *S.multipunctatus* (Pellegrin, 1931) and *S.cyphotergous* Dai, 1988. This group is characterized by having a humpback behind the head and pectoral fins with their adpressed tips exceeding pelvic-fin insertions (Zhao and Zhang 2009). With ongoing discoveries in the genus *Sinocyclocheilus*, the *S.cyphotergous* group has expanded to include a total of 17 species (Zhao and Zhang 2009; Lan et al. 2017; Jiang et al. 2019; Mao et al. 2021, 2022; Jiang et al. 2023; Luo et al. 2023; Fan et al. 2024; Shao et al. 2024). Recently, Shao et al. (2024) redefined a new group within the *S.cyphotergous* group, termed the *S.cyphotergous*-*S.multipunctatus* group. This new group currently includes five species, namely *S.multipunctatus*, *S.cyphotergous*, *S.punctatus* (Lan et al. 2017), *S.sanxiaensis* (Jiang et al. 2019), and *S.guiyang* (Shao et al. 2024). Characters typical for the *S.cyphotergous*-*S.multipunctatus* group are a humpback immediately behind the head, short barbels, and a deep head (Shao et al. 2024). These characters are shared with a new species under description in the present study.

Field surveys of cavefish, conducted into the karst area of the Dabang-He tributary to the Beipan-Jiang in Zhenning County, Anshun City, Guizhou Province, China in the spring of 2022 and the summer of 2023, yielded eight specimens referrable to *Sinocyclocheilus*. The integration of molecular and morphological evidence indicated that they represent a novel species of the genus, herein described as *S.zhenningensis* sp. nov. This study is the first to report on the occurrence of the *S.cyphotergous* group in the Beipan-Jiang. The discovery of this new species may contribute to further research on cavefish and their conservation.

## ﻿Material and methods

### ﻿Specimen collection

During a cavefish survey in southern China on 5 January 2022 and 8–9 July 2023, eight specimens of *Sinocyclocheilus* were collected in Biandanshan Town, Zhenning County, Anshun City, Guizhou Province, China. After the anesthesia of three freshly collected samples, muscle tissue was taken and preserved in absolute ethyl alcohol at −20 °C for molecular analysis. Their vouch specimens and the remaining five specimens were fixed in 10% formalin and then transferred and stored in 75% ethanol for morphometric analysis. All caught specimens are housed in the collection of the School of Life Sciences, Guizhou Normal University, Guiyang City, Guizhou Province, China.

### ﻿DNA extraction, PCR amplification, and sequencing

The standard high-salt method (Aljanabi and Martinez 1997) was employed to extract genomic DNA from the sampled muscle tissues. The mitochondrial cytochrome *b* (*Cytb*) gene, which is a widely used and crucial molecular marker for species identification for *Sinocyclocheilus*, was utilized to determine the genetic relationship of the novel species to other closely related species. The primer pairs L14724 (5′–CGAAGCTTG ATATGAAAAACCATCGTTG–3′) and H15915 (5′–ACCTCCGATCTYCGGATTAC AAGAC–3′) (Zhao et al. 2006; Luo et al. 2023) were used to amplify the *Cytb* gene by polymerase chain reaction (PCR).

The PCR amplification was carried out in a reaction volume of 35 μL and included 17.5 μL of 2×Taq MaxterMix (Dye), 1 μL of each primer (L14724 and H15915), 1 μL of DNA template and 14.5 μL ddH_2_O. The PCR program was as follows: an initial denaturation step at 95 °C for 5 min; denaturation at 95 °C for 30 s; annealing at 47.6 °C for 30 s, and extension at 72 °C for 70 s; 35 cycles of denaturation to extension; and a final extension at 72 °C for 10 min. The PCR products were examined by 1% agarose gel electrophoresis. The samples with clear bands corresponding to the target size were subsequently sent to Shanghai Biotechnology Corporation for bidirectional sequencing. All sequences have been submitted to GenBank for deposition (Table 1).

**Table 1.** GenBank accession numbers and references of all species included in the phylogenetic analysis of the *Sinocyclocheilus* cavefish.

Species	GenBank accession No.	References
*Sinocyclocheilusaltishoulderus*	AB196442	Zhao et al. 2006
*Sinocyclocheilusanatirostris*	AY854708	Xiao et al. 2005
*Sinocyclocheilusangularis*	MW362289	Luo and Zhang 2021
*Sinocyclocheilusangustiporus*	MZ636515	Wen et al. 2021
*Sinocyclocheilusanophthalmus*	AY854716	Xiao et al. 2005
*Sinocyclocheilusanshuiensis*	KR069120	He et al. 2016
*Sinocyclocheilusaquihornes*	OQ718393	Luo et al. 2023
*Sinocyclocheilusbicornutus*	AY854732	Xiao et al. 2005
*Sinocyclocheilusbrevibarbatus*	OQ718394	Luo et al. 2023
*Sinocyclocheilusbrevifinus*	OQ718395	Luo et al. 2023
*Sinocyclocheilusbrevis*	OQ718396	Luo et al. 2023
*Sinocyclocheiluscyphotergous*	AY854711	Xiao et al. 2005
*Sinocyclocheilusdonglanensis*	AB196440	Zhao et al. 2006
*Sinocyclocheilusflexuosdorsalis*	OQ718397	Luo et al. 2023
*Sinocyclocheilusfurcodorsalis*	AY854709	Xiao et al. 2005
*Sinocyclocheilusgracilicaudatus*	OQ718398	Luo et al. 2023
*Sinocyclocheilusgrahami*	AY854696	Xiao et al. 2005
*Sinocyclocheilusguanyangensis*	OQ718399	Luo et al. 2023
*Sinocyclocheilusguilinensis*	OQ718400	Luo et al. 2023
*Sinocyclocheilusguishanensis*	AY854722	Xiao et al. 2005
*Sinocyclocheilusguiyang*	OR141734	Shao et al. 2024
*Sinocyclocheilushuaningensis*	AY854718	Xiao et al. 2005
*Sinocyclocheilushuangtianensis*	OQ718401	Luo et al. 2023
*Sinocyclocheilushuanjiangensis*	OQ718402	Luo et al. 2023
*Sinocyclocheilushugeibarbus*	OQ718403	Luo et al. 2023
*Sinocyclocheilushuizeensis*	NC044072	Xu et al. 2019
*Sinocyclocheilushyalinus*	AY854721	Xiao et al. 2005
*Sinocyclocheilusjii*	AY854728	Xiao et al. 2005
*Sinocyclocheilusjiuxuensis*	AY854736	Xiao et al. 2005
*Sinocyclocheiluslateristriatus*	AY854707	Xiao et al. 2005
*Sinocyclocheiluslingyunensis*	AY854692	Xiao et al. 2005
*Sinocyclocheiluslongibarbatus*	AY854714	Xiao et al. 2005
*Sinocyclocheiluslongicornus*	MZ634123	Xu et al. 2023
*Sinocyclocheilusmacrocephalus*	AY854684	Xiao et al. 2005
*Sinocyclocheilusmacrolepis*	AY854729	Xiao et al. 2005
*Sinocyclocheilusmacrophthalmus*	AY854735	Xiao et al. 2005
*Sinocyclocheilusmaculatus*	EU366193	Li et al. 2008
*Sinocyclocheilusmalacopterus*	AY854697	Xiao et al. 2005
*Sinocyclocheilusmashanensis*	OQ718404	Luo et al. 2023
*Sinocyclocheilusmaitianheensis*	AY854710	Xiao et al. 2005
*Sinocyclocheilusmicrophthalmus*	AY854690	Xiao et al. 2005
*Sinocyclocheilusmultipunctatus*	AY854713	Xiao et al. 2005
*Sinocyclocheilusoxycephalus*	AY854685	Xiao et al. 2005
*Sinocyclocheiluspunctatus*	OQ718405	Luo et al. 2023
*Sinocyclocheiluspurpureus*	EU366194	Li et al. 2008
*Sinocyclocheilusqiubeiensis*	MF324975	Chen et al. 2018
*Sinocyclocheilusqujingensis*	AY854719	Li et al. 2008
*Sinocyclocheilusrhinocerous*	AY854720	Xiao et al. 2005
*Sinocyclocheilusronganensis*	KX778473	Luo et al. 2017
*Sinocyclocheilussanxiaensis*	MN106258	Jiang et al. 2019
*Sinocyclocheilussimengensis*	OQ718406	Luo et al. 2023
*Sinocyclocheilustianlinensis*	OQ718407	Luo et al. 2023
*Sinocyclocheilustingi*	AY854701	Xiao et al. 2005
*Sinocyclocheiluswenshanensis*	OQ718408	Luo et al. 2023
*Sinocyclocheiluswumengshanensis*	NC039769	Chen et al. 2017
*Sinocyclocheilusxichouensis*	OQ718409	Luo et al. 2023
*Sinocyclocheilusxingyiensis*	ON573218	Luo et al. 2023
*Sinocyclocheilusxunlensis*	EU366190	Li et al. 2008
*Sinocyclocheilusyangzongensis*	AY854726	Xiao et al. 2005
*Sinocyclocheilusyimenensis*	EU366191	Li et al. 2008
*Sinocyclocheilusyishanensis*	AB196443	Zhao et al. 2006
*Sinocyclocheiluszhenfengensis*	MW014317	Wen et al. 2021
*Sinocyclocheiluszhenningensis* ZN-2	PQ139368	This study
*Sinocyclocheiluszhenningensis* ZN-3	PQ139369	This study
*Sinocyclocheiluszhenningensis* ZN-8	PQ139367	This study
*Schizothoraxmacropogon*	AY954266	He and Chen 2006
*Cyprinuscarpio*	DQ868875	Tsipas et al. 2009

### ﻿Morphological comparisons

The morphometric and meristic data was collected, analyzed and characterized following previous research conducted on *Sinocyclocheilus* species (Zhao et al. 2006). For comparison, specimens of the two closest related epigean species, *S.multipunctatus* and *S.punctatus* of the *S.cyphotergous*-*S.multipunctatus* group, were also evaluated morphologically. Information on the specimen identities is given in the “Comparative materials” section.

All specimens were measured using an electronic Digital Vernier caliper (IP54 protection type) with a 0.01 mm precision, and all the measurements were rounded to one decimal place (Table 2). Morphological measurements are presented as a percentage of the standard length (SL), also rounded up to the nearest millimeter in the computed result. The computed tomography (CT) photographs of the holotype were procured with a micro-CT scanner (Bruker SkyScan 1276) to observe the osteological characters and all the vertebrae of the novel species were counted.

**Table 2.** Morphometric measurements of the holotype and seven paratypes of *Sinocyclocheiluszhenningensis* sp. nov. (*N* = 8).

Morphometric information	Holotype	Paratypes (*N* = 7)						
	ZN-7	ZN-1	ZN-2	ZN-3	ZN-4	ZN-5	ZN-6	ZN-8
Total length (TL, mm)	123.5	175.3	116.6	116.4	106.5	79.1	94.2	122.7
Standard length (SL, mm)	98.5	142.1	89.9	92.4	82.9	59.3	74.3	95.8
Head length (HL, mm)	28.7	40.1	26.2	26.6	25.0	18.1	22.7	28.2
% **SL**								
Body depth	29.3	26.8	30.1	26.5	29.2	29.7	27.1	28.7
Pre-dorsal length	57.2	53.6	58.1	57.4	56.9	58.2	56.7	54.1
Dorsal-fin base length	13.8	12.6	13.5	15.6	15.2	16.2	14.9	15.0
Dorsal-fin length	19.4	18.4	23.0	21.8	24.1	27.8	23.1	20.3
Pre-anal length	72.3	70.9	75.0	72.9	74.3	74.5	70.4	71.4
Anal-fin base length	8.2	7.9	9.3	9.3	9.3	8.6	8.7	9.1
Anal-fin length	17.1	17.2	17.0	18.2	17.0	17.9	18.3	16.8
Pre-pectoral length	28.9	27.3	27.5	29.6	30.7	31.4	30.6	29.5
Pectoral-fin base length	4.3	4.1	4.1	3.9	4.2	4.7	3.9	4.0
Pectoral-fin length	19.4	20.7	19.6	18.9	20.7	19.9	20.6	20.3
Pre-pelvic length	53.3	51.5	56.1	51.8	53.7	55.0	52.6	52.1
Pelvic-fin base length	5.5	4.8	4.8	4.8	4.6	4.2	5.2	4.4
Pelvic-fin length	16.6	16.6	17.0	16.6	17.4	18.9	15.9	17.2
Caudal peduncle length	20.2	17.1	19.6	16.7	20.2	18.7	20.3	15.7
Caudal peduncle depth	11.0	10.0	10.2	9.7	9.5	9.8	10.5	10.1
Head length	29.1	28.2	29.2	28.8	30.1	30.5	30.6	29.4
Head depth	17.4	17.3	17.6	17.3	18.1	18.9	17.0	17.7
Head width	15.2	15.7	16.5	14.6	15.7	15.3	14.9	15.6
Snout length	8.7	9.3	8.6	7.3	6.9	8.6	7.8	8.6
Eye-ball diameter	5.8	4.6	6.3	5.8	6.3	6.6	6.6	5.7
Eye diameter	7.5	5.8	7.3	6.9	8.0	7.3	8.2	7.4
Inter-orbital width	9.8	12.3	11.8	10.0	9.3	8.8	9.4	9.6
Pre-nostril distance	6.2	5.3	5.2	4.6	5.0	5.2	6.2	5.8
Distance between posterior nostrils	5.7	4.9	4.2	4.0	4.1	5.4	6.1	4.3
Upper jaw length	6.9	6.8	5.5	5.1	5.7	6.2	7.1	7.8
Lower jaw length	6.7	6.1	4.8	4.6	5.3	5.6	6.6	6.2
Mouth width	7.2	8.2	7.7	7.4	7.5	7.8	6.9	7.4
Maxilla barbel length	7.5	8.7	8.0	6.5	8.7	5.6	7.4	6.4
Rictal barbel length	8.5	9.4	8.7	8.2	9.2	6.6	8.2	7.9
**Meristic information**								
Dorsal-fin rays	iii-8	iii-8	iii-8	iii-8	iii-8	iii-8	iii-8	iii-8
Pectoral-fin rays	i-15	i-15	i-15	i-15	i-15	i-15	i-15	i-15
Pelvic-fin rays	ii-8	ii-8	ii-8	ii-8	ii-8	ii-8	ii-8	ii-8
Anal-fin rays	iii-5	iii-6	iii-6	iii-6	iii-6	iii-6	iii-5	iii-5
Principal caudal-fin rays	18i-17	18i-17	18i-17	18i-17	18i-17	18i-17	18i-17	18i-17
Perforated lateral-line scales	47.0	47.0	45.0	45.0	44.0	42.0	44.0	47.0

### ﻿Phylogenetic analyses

To infer the phylogenetic relationships of the new species, the available *Cytb* sequences of *Sinocyclocheilus* were retrieved from the GenBank database (https://www.ncbi.nlm. nih.gov/) (Table 1). *Schizothoraxmacropogon* Regan, 1905 and *Cyprinuscarpio* Linnaeus, 1758, were selected as the outgroups as done in previous studies (Zhao and Zhang 2009; Luo et al. 2023) (Table 2). The obtained raw sequences were aligned and manually corrected using the default settings of CLUSTAL-W’s codon alignment mode in MEGA11 (Tamura et al. 2021). Additionally, MAFFT (Strategy elect L-INS-i) in PhyloSuite v. 1.2.3 (Zhang et al. 2020) was used to align the sequences for the final dataset used for phylogenetic analyses. The final dataset consisted of sequences from 65 species of *Sinocyclocheilus* and their interspecific phylogenetic relationships were inferred by Bayesian inference (BI) using MrBayes v. 3.2.7 (Ronquist et al. 2012). ModelFinder v. 2.2.0 (Kalyaanamoorthy et al. 2017) was used to select the best-fit model using the corrected Akaike Information Criterion (AICc), and the GTR+F+I+G4 model was chosen. Four Markov Chain Monte Carlo (MCMC) chains were used, consisting of three heated chains and one cold chain. Commencing from a randomly generated tree, the Markov chains iterated for five million generations, with samples collected at every 100 generations. And then ensuring the average standard deviation of split frequencies was < 0.01, the first 25% of samples were discarded as burn-in. The BI phylogenetic trees were viewed and modified using the Interactive Tree of Life (iTOL) (https://itol.embl.de/) (Letunic and Bork 2021). Genetic distances among species within the *S.cyphotergous*-*S.multipunctatus* subclade, including the new species, were calculated in MEGA11 (Tamura et al. 2021), based on the Kimura 2-parameter (K2P) model (Kimura 1980) and are reported as percentages with one decimal place.

## ﻿Results

### ﻿Taxonomy

#### ﻿ Sinocyclocheilus zhenningensis sp. nov.

Taxon classificationAnimaliaCypriniformesCyprinidae

616BC856-365E-5603-9820-5E195DEA6521

https://zoobank.org/D5D2598D-C4EF-4F17-8FA0-5DBDB4A7903A

**Figure 1.**
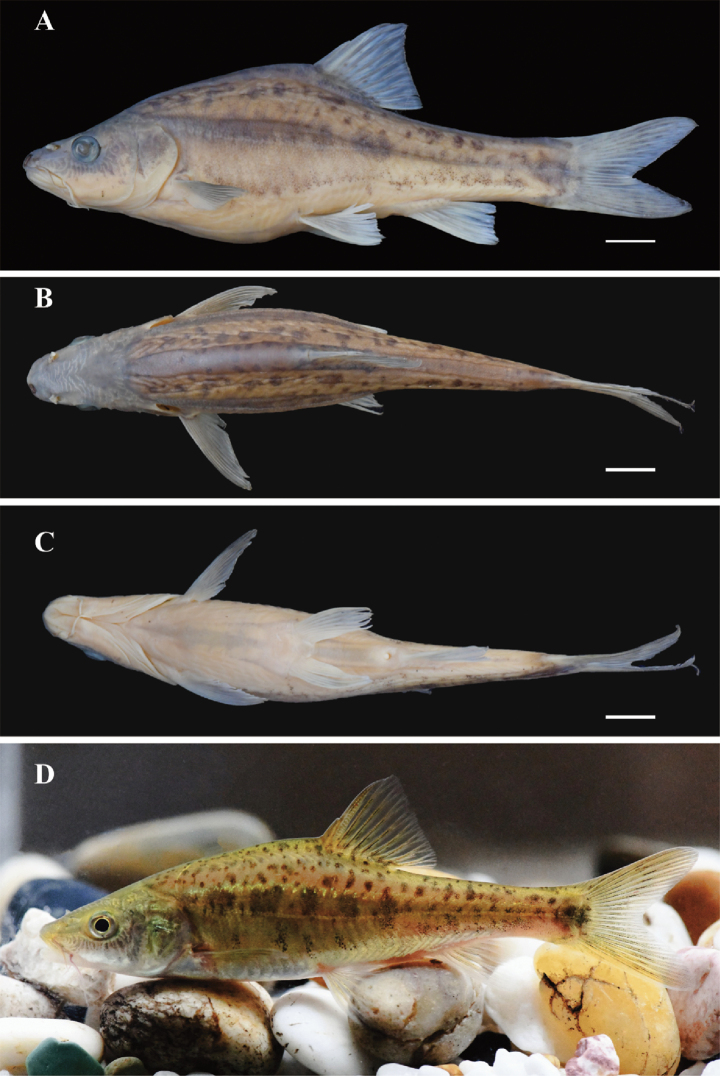
*Sinocyclocheiluszhenningensis* (GZNUSLS202201109, ZN-7), holotype, 123.5 mm SL; lateral (**A**), dorsal (**B**), and ventral (**C**) view of body. Live photo (**D**). Scale bar: 10 mm.

**Figure 2.**
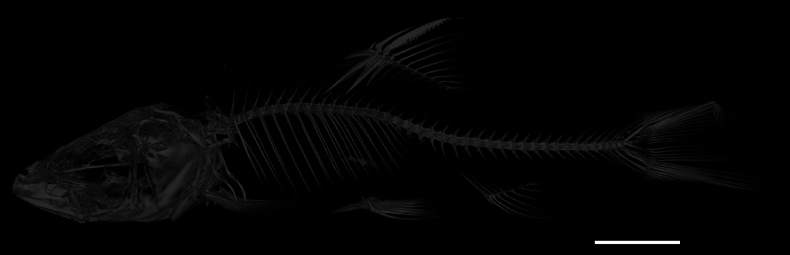
*Sinocyclocheiluszhenningensis* sp. nov., paratype (GZNUSLS202201108, ZN-6), 94.2 mm SL. Micro-CT scanning image of paratype; lateral view.

Figs 1 , 2 , Table 2

##### Holotype.

GZNUSLS202201109, one specimen (ZN-7), 98.5 mm SL; Dabang-He of Beipan-Jiang at Kongma Village (26°14'24"N, 105°36'49"E), Biandanshan Town, Zhenning County, Anshun City, Guizhou Province, China; collected by Renyi Zhang and Qi Luo on 5 January 2022.

##### Paratype.

GZNUSLS202307047–51, five specimens, 59.3–142.1 mm SL, Dabang-He of Beipan-Jiang at Aozizhai village (23°11'39"N, 106°25'53"E), Biandanshan Town, Zhenning County, Anshun City, Guizhou Province, China; collected by Renyi Zhang, Leishan Wang, Huan Cheng, and Renrong Huang in 8–9 July 2023. GZNUSLS202201060, GZNUSLS202201108, two specimens, 74.3–95.8 mm SL; all other data same as holotype.

##### Etymology.

The specific epithet, used as a noun in apposition, is derived from the name of the type locality (Zhenning County). We propose this new cavefish with the English common name Zhenning Golden-lined Barbel and the Chinese common name Zhèn níng Jīn Xiàn Bā (镇宁金线鲃).

##### Diagnosis.

The new species is assigned to the *S.cyphotergous*-*S.multipunctatus* group of the genus *Sinocyclocheilus* based on the *Cytb* genes phylogenetic analysis and morphological characters. *Sinocyclocheiluszhenningensis* sp. nov. can be differentiated from other known congeners by the following combination of morphological characters: (1) normal eyes (5.8%–8.2% SL) (vs absence or degeneration), presence of pigmentation (vs absence); no horn structure (vs presence), with a humpback (vs absence); (2) pelvic-fin rays ii-8 (vs i-8), dorsal-fin rays iii-8, stiff and stout last unbranched ray (vs soft and no serrations); (3) body depth normal (26.5%–30.1% SL) and covered with irregular black spots (vs no irregular black spots), body scaled, but scales buried beneath the skin (vs absence or degeneration); (4) lateral line complete (vs absence or degeneration), flat and slightly curved at the bottom of the corresponding dorsal fin, 42–47 lateral line pored scales (vs > 47); and (5) pectoral-fin length short, not reaching anterior base of pelvic fin (vs pectoral-fin length reaching or exceeding anterior base of pelvic fin).

##### Description.

General body appearance is given in Fig. 1. Measurement distances and meristic counts are shown in Table 2. Dorsal fin with three unbranched and eight-branched rays; pelvic fin with two unbranched and eight-branched anal-fin rays iii-6(5); pectoral fin with one unbranched and five-branched rays. Fifteen pre-dorsal vertebrae; total vertebrae 38 (*N* = 1); 9–10 inner-gill rakers on first gill arch (in eight specimens). Body scaled, but scales buried beneath the skin, covered with irregular black spots. Lateral line complete from uppermost point of gill slit to caudal-fin base, with 42–47 pored scales.

Body moderately elongated and laterally compressed, deeper than wide, with maximum depth immediately at dorsal-fin origin. Dorsal profile gently arched from snout tip to dorsal-fin origin, Pelvic-fin insertion anterior to vertical through dorsal-fin origin. ventral profile of head slightly curved, rounded between pectoral- and pelvic-fin insertion, and slightly concave or straight from pelvic-fin insertion to caudal-fin base.

Head triangular and compressed slightly, longer than deep, deeper than wide. Two pairs of nostrils, distance between tip of snout and anterior margin of orbit, exactly half of distance between two nostrils. Mouth subterminal and horse shoe-shaped; upper jaw slightly longer than lower jaw. Two pairs of barbels; rostral pair underneath anterior nostril, short and only slightly beyond posterior edge of posterior nostrils; maxillary barbels short, extending beyond anterior edge of eye, not reaching posterior-most edge of gill cover. Eye large, rounded. Gill rakers well-developed, with 9–10 in first branchial arch.

Dorsal fin with three unbranched and eight branched rays; last unbranched ray rigid at its base and gradually becoming softer toward the tip; with prominent serrations along its posterior edge; origin approximately halfway between tip of snout and caudal-fin base. Pectoral fin short, with one unbranched ray and 15 branched rays, inserted immediately under posterior-most edge of gill cover, tip of adpressed rays approaching to pelvic-fin insertion. Pelvic fins relatively short, with two unbranched rays and eight branched rays; inserted below dorsal-fin origin; tip of adpressed rays not extending to anus. Anal fin with three unbranched rays and 5 or 6 branched rays; origin slightly posterior to anus, and closer to pelvic-fin insertion than to caudal-fin base. Caudal fin deeply forked.

##### Coloration.

In freshly collected individuals, ground color of body golden yellow or brown dorsally and laterally, and pale whitish on belly. A distinct gold line above lateral line, anteriorly lighter, and many black bars of various sizes scattered over dorsum. Dark yellow band along middle of flank before anal-fin origin, anteriorly deeper and becoming narrower backwards to end in a large rounded black bar on caudal-fin base, with five squarish brown blotches immediately above and five indistinct vertical bars below lateral line before anal-fin origin, and from then many small black bars on nearby area close to lateral line. Dorsal fin hyaline with pigments along branched rays, pectoral and pelvic fins white, and caudal fin grey and white, with black spots scattering on the middle of caudal-fin lobes to form an indistinct W-shaped mark.

In formalin-preserved individuals, dorsum and flank dark grey, chest and abdomen white with yellow, grey-white dorsal and caudal fins, pelvic- and pectoral-fins white. The preserved specimens in alcohol, showed distinct markings on their bodies, characterized by black spots of varying size, generally located exclusively above the lateral line, while fewer or no black spots below the lateral line were observed in the soaked specimens.

##### Distribution and habitat.

**Figure 3.**
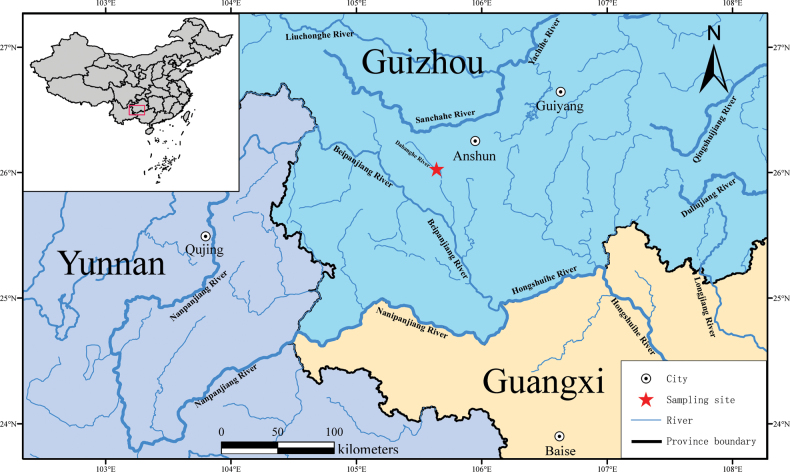
The sampling site of *Sinocyclocheiluszhenningensis* sp. nov.

**Figure 4.**
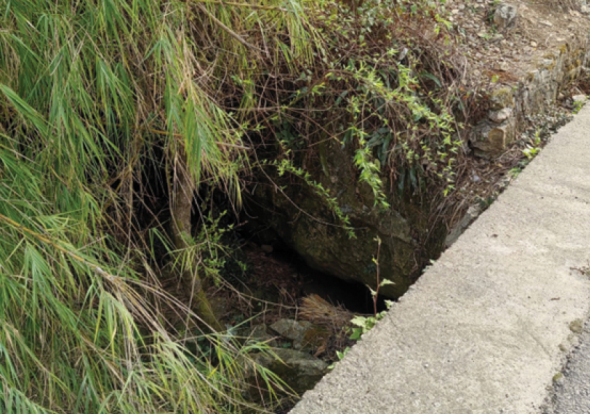
The epigean entrance of the karst cave and subterranean river at the type locality of *S.zhenningensis* sp. nov., located in Zhenning County, Anshun City, Guizhou Province, China.

The holotype and paratype specimens were obtained from a karst cave in a subterranean river. The coexisting fishes include *Pterocryptisanomala* Herre, 1933 and an unidentified species of *Triplophysa*. There are many karst landforms in the area of the Dabang-He, a tributary of the Beipan-Jiang (Fig. 3). The entrance of the cave is less than 1.5 meters in width. The subterranean river drains into the Dabang-He (Fig. 4).

##### Genetic evidence from phylogenetic analysis.

**Figure 5.**
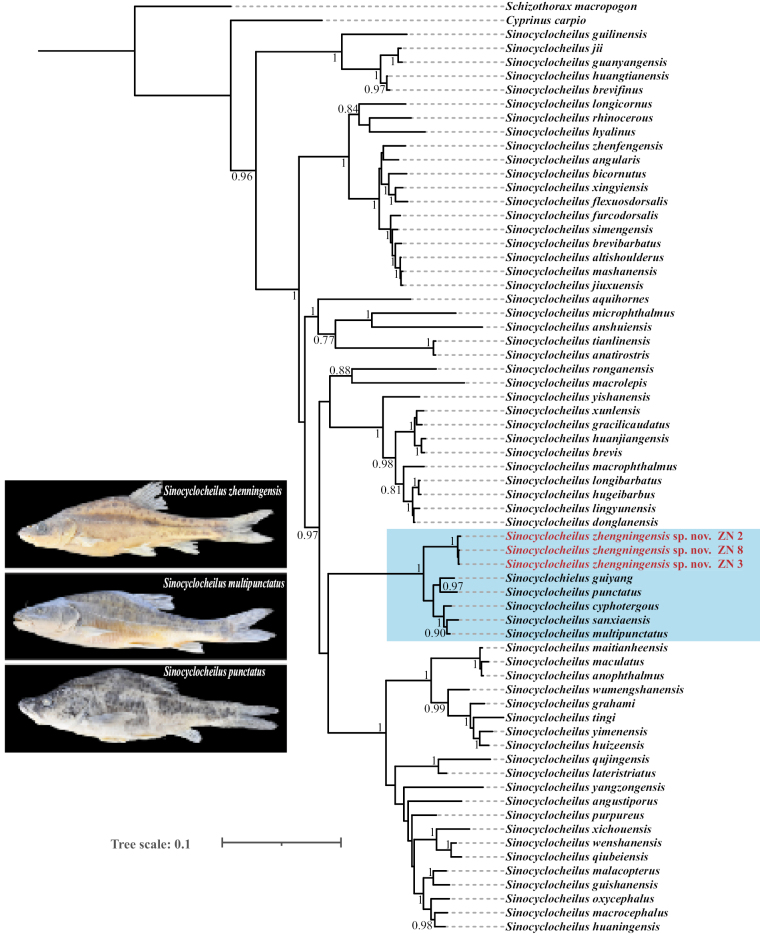
The Bayesian inference (BI) tree of *Sinocyclocheilus* species based on the mitochondrial *Cytb* gene. Bayesian posterior probabilities are indicated at the nodes; only values exceeding 0.75 were displayed. The light blue area indicates the *S.cyphotergous*-*S.multipunctatus* group.

The *Cytb* dataset, 1140 bp in length, was used to reconstruct phylogenetic relationships of sampled species of *Sinocyclocheilus*. Three amplified *Cytb* sequences of this new species have been submitted to GenBank (Table 1). The analysis revealed that *S.zhenningensis* sp. nov. is sister to the *S.cyphotergous*-*S.multipunctatus* subclade with strong support (bootstrap values = 1) (Fig. 5). In the BI phylogenetic tree, the *S.cyphotergous*-*S.multipunctatus* group (Shao et al. 2024) is nested within the *S.cyphotergous* group (Zhao and Zhang 2009) (Fig. 5). The genetic distances of *S.zhenningensis* sp. nov. ranged from 3.8% (with *S.cyphotergous*) to 4.6% (with *S.punctatus*) in the *S.cyphotergous*-*S.multipunctatus* group (Table 3). The minimum genetic distance between *S.zhenningensis* sp. nov. and *S.cyphotergous* (Table 3) exceeds the threshold of 2% mitochondrial DNA variation, and both can be clearly distinguished morphologically.

**Table 3.** The K2P genetic distance (%) in *Cytb* sequences between species in the *S.multipunctatus*-*S.cyphotergous* group.

Order	Species	1	2	3	4	5	6	7	8
1	*S.zhenningensis* ZN-8	0.0							
2	*S.zhenningensis* ZN-2	0.2	0.0						
3	*S.zhenningensis* ZN-3	0.0	0.2	0.0					
4	*S.multipunctatus*	4.1	4.3	4.1	0.0				
5	*S.punctatus*	4.4	4.6	4.4	2.7	0.0			
6	*S.sanxiaensis*	4.3	4.5	4.3	1.1	2.8	0.0		
7	*S.cyphotergous*	3.8	4.0	3.8	0.9	2.9	1.5	0.0	
8	*S.guiyang*	4.0	4.2	4.0	2.5	2.3	3.3	2.7	0.0

## ﻿Discussion

Integrative analyses of morphological and molecular evidence confirmed the specific status of *S.zhenningensis* sp. nov. within the well-defined *S.cyphotergous*-*S.multipunctatus* group, a distinct clade in the cavefish genus *Sinocyclocheilus* (Shao et al. 2024). In previous studies, the monophyly of this group has been confirmed (Wen et al. 2022; Jiang et al. 2023; Luo et al. 2023; Xu et al. 2023; Shao et al. 2024). The discovery of the new species under description brings the total number of valid *Sinocyclocheilus* species to 81.

In the *S.cyphotergous*-*S.multipunctatus* group, *S.cyphotergous*, *S.sanxiaensis*, and *S.guiyang* are different from *S.zhenningensis* sp. nov., with all lacking irregular black spots and exhibiting eye absence or degeneration. Additionally, *S.cyphotergous* is characterized by a humpback profile with a fleshy dorsal projection (Zhao and Zhang 2009), making it easily distinguishable from *S.zhenningensis* sp. nov. The two more similar species to *S.zhenningensis* sp. nov. (all with a humpback, a deep head, short barbles, normal eyes, presence of pigmentation, and body depth resemblance with irregular black spots) are *S.multipunctatus* (Wu and Lv 1983; Zhao and Zhang 2009) and *S.punctatus* (Lan et al. 2017). *Sinocyclocheiluszhenningensis* sp. nov. further differs from *S.multipunctatus* and *S.punctatus* as follows: a body depth (26.5%–30.1% SL) greater than that of *S.multipunctatus* (20.0%–24.7% SL), but not less than that of *S.punctatus* (31.0%–34.7% SL). It has a normal eye-ball diameter (4.6%–6.6% SL vs *S.punctatus* 7.6%–11.4% SL), an inter-orbital width of 8.8%–12.3% SL (vs *S.punctatus* 4.9%–6.0% SL), and a distance between the posterior nostrils of 4.0%–6.1% SL (vs *S.punctatus* 8.9%–10.6% SL), which can distinguish it from *S.multipunctatus* and *S.punctatus*. Additionally, *S.zhenningensis* sp. nov. has a nearly straight lateral line, is slightly curved ventrally, has 42–47 lateral-line scales, and can be distinguished from *S.multipunctatus* and *S.punctatus* by 50–57 and 51–71 lateral-line scales. Its two unbranched pelvic-fin rays, rictal barbels, and body depth in percentage of standard length (SL) can also aid in distinguishing this Zhenning golden fish from *S.punctatus* and *S.multipunctatus* (Table 4).

**Table 4.** Morphological comparison of *Sinocyclocheiluszhenningensis*, *S.multipunctatus*, and *S.punctatus*.

Morphometric information	*S.zhenningensis* (*N* = 8)		*S.multipunctatus* (*N* = 4)		*S.punctatus* (*N* = 7)	
	range	Mean ± SD	range	Mean ± SD	range	Mean ± SD
Total length (TL, mm)	79.1–175.3	114.0 ± 28.1	77.5–109.2	93.1 ± 13.1	100.5–176.9	129.5 ± 29.8
Standard length (SL, mm)	59.3–142.1	89.4 ± 24.0	61.0–86.4	73.4 ± 10.5	78.4–139.9	101.1 ± 24.8
Head length (HL, mm)	18.1–40.1	26.4 ± 6.3	18.1–25.6	22.2 ± 3.2	14.8–29.0	32.5 ± 7.8
% **SL**						
Body depth	26.5–30.1	28.4 ± 1.4	20.0–24.7	22.0 ± 2.1	31.0–34.7	32.9 ± 1.2
Pre-dorsal length	53.6–58.2	56.5 ± 1.7	52.6–56.1	54.7 ± 1.6	57.3–62.2	59.6 ± 1.6
Dorsal-fin base length	12.6–16.2	14.6 ± 1.2	14.7–15.9	15.5 ± 0.6	13.8–15.4	14.5 ± 0.6
Dorsal-fin length	18.4–27.8	22.1 ± 3.0	21.1–23.1	22.2 ± 0.8	19.7–26.1	22.9 ± 2.2
Pre-anal length	70.4–75.0	72.7 ± 1.8	72.3–73.7	73.3 ± 0.7	71.9–75.9	74.2 ± 1.6
Anal-fin base length	7.9–9.3	8.8 ± 0.5	8.2–9.3	8.8 ± 0.5	7.8–8.8	8.4 ± 0.4
Anal-fin length	16.8–18.3	17.4 ± 0.6	17.3–18.0	17.6 ± 0.3	16.7–20.7	18.7 ± 1.4
Pre-pectoral length	27.3–31.4	29.4 ± 1.5	30.7–31.9	31.4 ± 0.5	30.6–33.8	32.1 ± 1.2
Pectoral-fin base length	3.9–4.7	4.1 ± 0.3	4.2–4.7	4.4 ± 0.2	3.9–4.9	4.3 ± 0.3
Pectoral-fin length	18.9–20.7	20.0 ± 0.7	18.6–19.1	18.8 ± 0.2	20.8–22.7	21.6 ± 0.7
Pre-pelvic length	51.5–56.1	53.2 ± 1.6	51.0–53.4	52.1 ± 1.1	52.2–57.8	54.7 ± 1.8
Pelvic-fin base length	4.2–5.5	4.8 ± 0.4	4.3–4.9	4.6 ± 0.3	3.6–4.9	4.4 ± 0.5
Pelvic-fin length	15.9–18.9	17.0 ± 0.9	16.4–17.4	17.0 ± 0.4	17.4–20.0	18.9 ± 0.9
Caudal peduncle length	15.7–20.3	18.5 ± 1.8	17.1–18.7	17.9 ± 0.7	17.4–19.7	18.5 ± 0.8
Caudal peduncle depth	9.5–11.0	10.1 ± 0.5	8.9–9.7	9.3 ± 0.4	10.7–12.7	11.8 ± 0.7
Head length	28.2–30.6	29.5 ± 0.8	29.6–31.1	30.3 ± 0.7	30.6–33.9	32.1 ± 1.4
Head depth	17.0–18.9	17.6 ± 0.6	16.8–17.9	17.4 ± 0.5	18.7–20.7	19.4 ± 0.8
Head width	14.6–16.5	15.4 ± 0.6	14.5–15.5	15.1 ± 0.4	13.8–14.9	14.2 ± 0.4
Snout length	6.9–9.3	8.2 ± 0.8	7.9–9.1	8.7 ± 0.6	8.6–9.3	8.9 ± 0.2
Eye-ball diameter	4.6–6.6	5.9 ± 0.7	6.0–7.7	6.8 ± 0.7	7.6–11.4	9.5 ± 1.4
Eye diameter	5.8–8.2	7.3 ± 0.7	7.2–8.3	7.8 ± 0.5	7.6–8.7	8.3 ± 0.4
Inter-orbital width	8.8–12.3	10.1 ± 1.3	8.2–9.3	8.8 ± 0.5	4.9–6.0	5.5 ± 0.4
Pre-nostril distance	4.6–6.2	5.4 ± 0.6	5.9–6.3	6.1 ± 0.2	5.5–6.6	5.9 ± 0.4
Distance between posterior nostrils	4.0–6.1	4.8 ± 0.8	4.2–4.6	4.4 ± 0.2	8.9–10.6	9.5 ± 0.6
Upper jaw length	5.1–7.8	6.3 ± 0.9	6.4–7.2	6.7 ± 0.4	8.1–9.5	8.5 ± 0.5
Lower jaw length	4.6–6.7	5.7 ± 0.8	6.0–6.7	6.2 ± 0.3	7.5–8.7	8.1 ± 0.4
Mouth width	6.9–8.2	7.5 ± 0.4	6.8–7.5	7.1 ± 0.3	4.9–6.6	5.8 ± 0.6
Maxilla barbel length	5.6–8.7	7.3 ± 1.1	5.8–7.2	6.7 ± 0.6	5.7–8.5	6.6 ± 1.0
Rictal barbel length	6.6–9.4	8.3 ± 0.9	6.5–8.2	7.6 ± 0.8	4.6–6.5	5.5 ± 0.7
**Meristic information**						
Dorsal-fin rays	iii-8		iii-8		iii-8	
Pectoral-fin rays	i-15		i-15(14)		i-14	
Pelvic-fin rays	ii-8		i-8		ii-8	
Anal-fin rays	iii-6(5)		iii-5		iii-5	
Principal caudal-fin rays	17		17		17	
Perforated lateral-line scales	42–47		50–57		51–71	

The new species occurrence in the Beipan-Jiang Basin also represents the first record of the *S.multipunctatus*-*S.cyphotergous* group in the Beipan-Jiang Basin. The *Sinocyclocheilusangularis* group members, *Sinocyclocheilusangularis* (Zheng & Wang, 1990), *S.bicornutus* (Wang & Liao, 1997), and *S.zhenfengensis* (Liu et al., 2018), are also found in the Beipan-Jiang Basin; the three species have horns and can be easily distinguished from *S.zhenningensis* sp. nov. Moreover, *S.xiejiahuai* (Fan et al., 2024), belonging to the *S.tingi* group, is also found in the Beipan-Jiang Basin. *Sinocyclocheiluszhenningensis* sp. nov. can be distinguished from *S.xiejiahuai* by, for example, the lateral-line scales (42–47 vs 74) and irregular markings on the body lateral (vs absence in *S.xiejiahuai*).

Based on the *Cytb* gene sequences from 65 species of the genus *Sinocyclocheilus*, we reconstructed a BI tree to infer the phylogenetic relationships of *S.zhenningensis* sp. nov. Our results are consistent with Luo et al. (2023) and Shao et al. (2024), where *S.zhenningensis* sp. nov. is categorized within the *S.cyphotergous*-*S.multipunctatus* group (Fig. 5). Exceeding the 2% mitochondrial DNA variation benchmark, often considered a signature of distinct species across numerous taxonomic groups (Avise and Walker 1999), and reflecting agreement with the genetic divergence criteria used to classify existing *Sinocyclocheilus*. The genetic differences between *S.zhenningensis* sp. nov. and its close relatives *S.punctatus* and *S.multipunctatus* exceeded 4.0% (Table 3), where genetic divergences between 3.8%–4.6% were calculated for the *S.multipunctatus*-*S.cyphotergous* group in this study. Importantly, morphometric and genetic distance analyses yielded consistent results.

Recent studies of new species of *Sinocyclocheilus* (Luo et al. 2023; Xu et al. 2023; Fan et al. 2024; Shao et al. 2024) suggest a significant lack of information regarding these mostly inaccessible cave environments in the subterranean waters of the Guizhou Province. Similarly, the discovery of this newly discovered fish species suggests that there may be numerous regions in Guizhou Province that are potential habitats for other undiscovered *Sinocyclocheilus* species. Additionally, the integration of morphological and molecular methodologies offers a potent strategy for effectively differentiating closely related *Sinocyclocheilus* species. The population of *S.zhenningensis* sp. nov. is known only from the type locality, and the population size of this species may be very small, requiring further research and possibly conservation efforts.

## ﻿Conclusion

In summary, based on morphological and molecular evidence, *S.zhenningensis* sp. nov. is recognized as a novel species of the genus *Sinocyclocheilus*. Despite the abundant diversity of fish in the subterranean waters across southern China, especially in the Guizhou Province, very little is known about these cave systems, their inhabitants and the diversity of fish living there. Therefore, this new species description of *Sinocyclocheilus* will hopefully spur further research and conservation of this unique habitat and its extraordinary fauna.

### ﻿Comparative materials

All specimens were collected from Guizhou. The measurements of the morphology are given in Table 4.

*Sinocyclocheilusmultipunctatus*, GZNUSLS202210022–25, four specimens, 67.1–97.2 mm SL, Huaxi County, Guiyang City, Guizhou Province, China; Yangtze River Basin.

*Sinocyclocheiluspunctatus*, GZNUSLS202008127–140, seven specimens were selected based on preserved morphology, 78.4–139.9 mm SL, Libo County, Qiannan Buyi and Miao Autonomous Prefecture of Guizhou, China; Pearl River Basin.

## Supplementary Material

XML Treatment for Sinocyclocheilus zhenningensis

## ﻿Additional information

### Author ORCIDs

Leishan Wang https://orcid.org/0009-0000-4802-945X

Renyi Zhang https://orcid.org/0000-0002-3647-1416

### Data availability

All of the data that support the findings of this study are available in the main text.
